# Copy Number Variation Mapping and Genomic Variation of Autochthonous and Commercial Turkey Populations

**DOI:** 10.3389/fgene.2019.00982

**Published:** 2019-10-29

**Authors:** Maria G. Strillacci, Erica Gorla, Angel Ríos-Utrera, Vicente E. Vega-Murillo, Moises Montaño-Bermudez, Adriana Garcia-Ruiz, Silvia Cerolini, Sergio I. Román-Ponce, Alessandro Bagnato

**Affiliations:** ^1^Department of Veterinary Medicine, Università degli Studi di Milano, Milano, Italy; ^2^Campo Experimental La Posta, INIFAP, Municipio de Medellín, Veracruz, Mexico; ^3^Centro Nacional de Investigación en Fisiología y Mejoramiento Animal, INIFAP, Auchitlán, Querétaro, Mexico

**Keywords:** *Meleagris gallopavo*, structural variation, copy number variant, genetic diversity, biodiversity

## Abstract

This study aims at investigating genomic diversity of several turkey populations using Copy Number Variants (CNVs). A total of 115 individuals from six Italian breeds (Colle Euganei, Bronzato Comune Italiano, Parma e Piacenza, Brianzolo, Nero d’Italia, and Ermellinato di Rovigo), seven Narragansett, 38 commercial hybrids, and 30 Mexican turkeys, were genotyped with the Affymetrix 600K single nucleotide polymorphism (SNP) turkey array. The CNV calling was performed with the Hidden Markov Model of PennCNV software and with the Copy Number Analysis Module of SVS 8.4 by Golden Helix^®^. CNV were summarized into CNV regions (CNVRs) at population level using BEDTools. Variability among populations has been addressed by hierarchical clustering (pvclust R package) and by principal component analysis (PCA). A total of 2,987 CNVs were identified covering 4.65% of the autosomes of the Turkey_5.0/melGal5 assembly. The CNVRs identified in at least two individuals were 362—189 gains, 116 losses, and 57 complexes. Among these regions the 51% contain annotated genes. This study is the first CNV mapping of turkey population using 600K chip. CNVs clustered the individuals according to population and their geographical origin. CNVs are known to be indicators also of adaptation, as some researches in different species are suggesting.

## Introduction

The domestication of the wild turkey appears to occur in Mexico between 200 B.C. and 700 A.D. (Crawford, 1992). The domesticated turkey has been introduced in Europe from Mexico and Central America starting in late 15^th^ century (Schorger, 1996) by the Spanish conquerors. The diffusion of the turkey population in the European territory was very fast, close to 50 km per year as indicated by Crawford (1992). The rapid diffusion in Europe was possibly facilitated because of their farming, as turkey was appreciated for its meat (Schorger, 1996). Then, since the 15^th^ century, the populations of European and Mexican turkey evolved independently for more than 500 years.

At present in Europe there is a clear differentiation in several turkey breeds, indicating that farmers and breeders have selected the turkey populations according to a directional goal for more than 5 centuries. Additionally, in the last 40 years, companies developed a structured breeding plan to produce commercial hybrids selected to maximize meat production^1^.

In this study six Italian autochthonous breeds [Colle Euganei (CoEu); Bronzato Comune Italiano (BrCI); Parma e Piacenza (PrPc); Brianzolo (BR); Nero d’Italia (NI) Ermellinato di Rovigo (ErRo)], the Narragansett, the Mexican turkey, and a hybrid population were considered to disclose genome structural variations in a wide dataset of individuals from differently evolved populations.

The selection operated by farmers in the past 5 centuries in the Italian populations determined the appearance of strong variation in plumage colors, in body size and weight, differentiating the populations in breeds (Cavalchini, 1983). This differentiation was possibly also facilitated by the geopolitical structure of Italy in middle ages, structured in a large number of small states with very limited exchange of goods and populations, making each turkey population genetically isolated from the others. Plumage of these breeds spans from totally black (Nero d’Italia) to white with black streaks (Ermellinato di Rovigo), while it is generally bronze like or with bronze reflection in all the other Italian populations. Body size is also showing a considerable difference among the Italian breeds with male weight spanning from 4.5 to 6.5 kg in the Brianzolo and reaching 12 kg in the Ermellinato di Rovigo (**Table 1**). Due to the fact that local farming occurred for centuries, it is expected that genetic bottleneck occurred in the Italian populations. The Mexican turkey population has historically been farmed as a backyard population without any directional selection for centuries, with a plumage very variable in its color and a weight close to 6 kg in males. In fact, in this population, there is not any structured selection program, while its genetic peculiarity is a strong argument in favor of its conservation (Utrera et al., 2016). In the farming system birds are free to migrate, facilitating the exchange of genetics across the country, favoring the genetic variability occurring in the population thus contributing to its morphological homogeneity irrespectively from the geographical location. The Narragansett breed (NARR) originated in Rhode Island and was recognized as a breed at the end of the 19^th^ century. The NARR was originally developed in Rhode Island by colonies returning to America from Europe in 16^th^ century, bringing back turkeys of the Norfolk Black breed and crossing them with the native American ones (Ekarius, 2007).

**Table 1:** Population name, sampling area, weight (kg) and plumage color of the turkey populations considered in the study.

Brianzolo (BR)*	Bronzato Comune Italiano (BrCI)*	Colle Euganei (CoEu)**	Ermellinato di Rovigo (ErRo)**
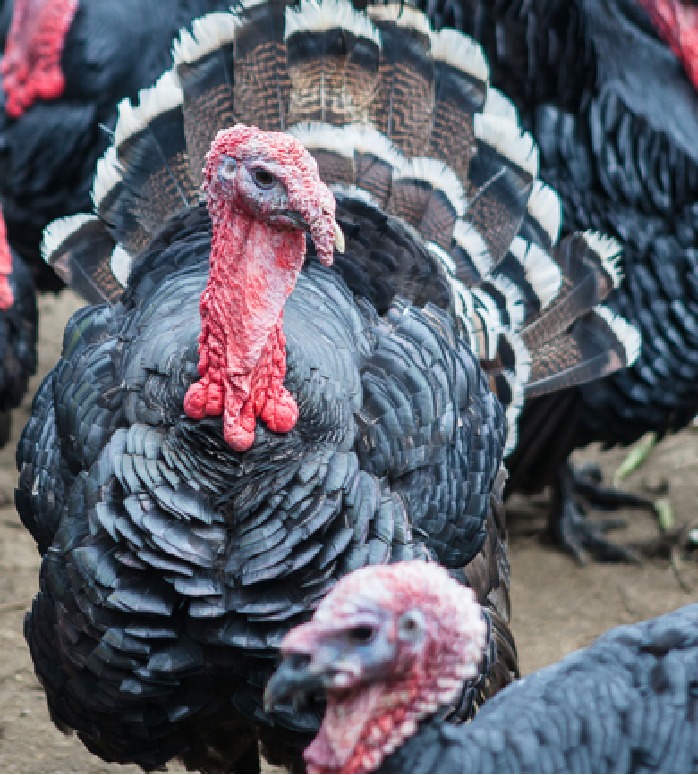 **Origin Area**: North Italy (Lombardia) **Weight (kg):** F:2.1-3.2; M: 4.5-6.4 **N. eggs/year:** 47 **Fertility:** 77-78% **Plumage:** Black, bronzed, reticulated gray (common), bronzed with white wings. **Description:** Early and disease-resistant bird. Rural breeding, numerical consistency extremely small.	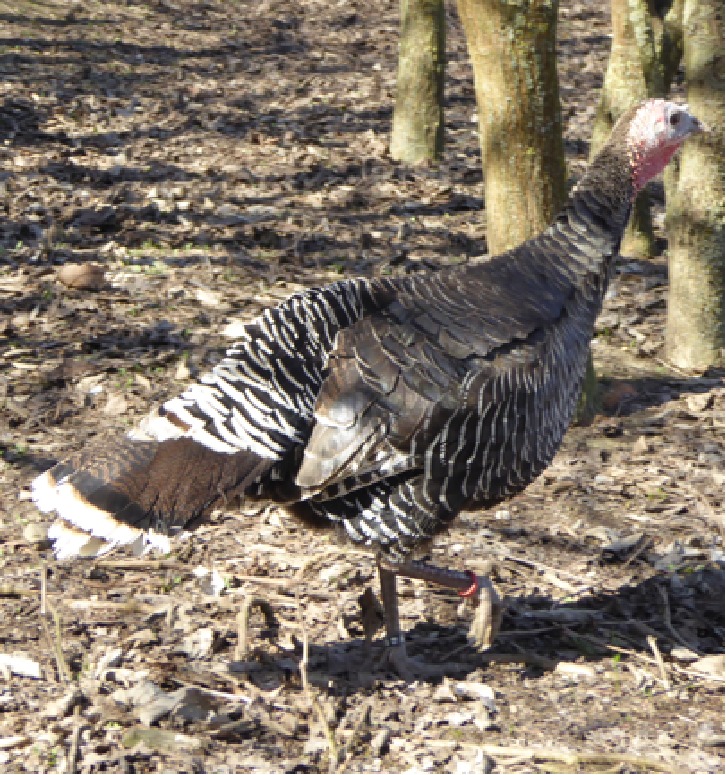 **Origin Area**: North-East Italy (Veneto) **Weight (kg):** F:3-3.5; M: 6-7 **N. eggs/year:** 70-100 **Fertility:** 92-93% **Plumage:** brilliant black with intense bronze reflections. **Description:** Rustic breed with a strong hatching attitude. Breeding in local areas.	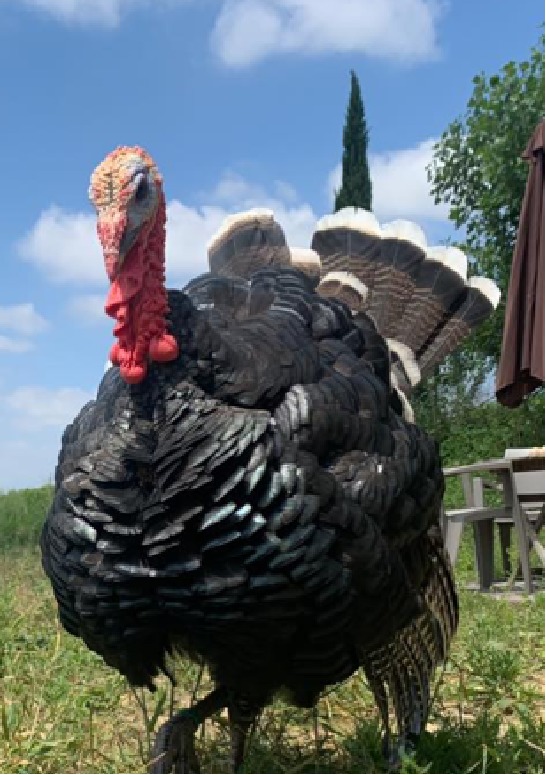 **Origin Area**: North-East Italy (Veneto) **Weight (kg):** F:3; M: 5 **N. eggs/year:** N/A **Fertility:** N/A **Plumage:** bronzed with metallic reflections. **Description:** Rustic breed with a strong hatching attitude. Local breeding, numerical consistency extremely small.	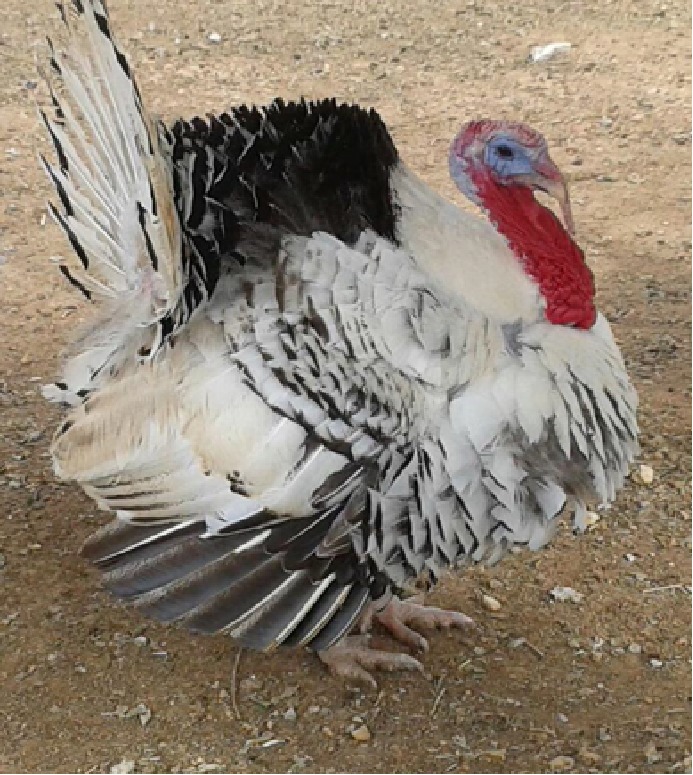 **Origin Area**: North-East Italy (Veneto) **Weight (kg):** F:4-6; M: 10-12 **N. eggs/year:** 70-80 **Fertility:** 86-92% **Plumage:** white with black streaks. **Description:** Rustic breed with slow growing excellent grazers. Breeding in local areas.
Nero Italiano (NI)*	Parma e Piacenza (PrPc)**	Mexican (MEX)***	Narragansett (NARR)**; §
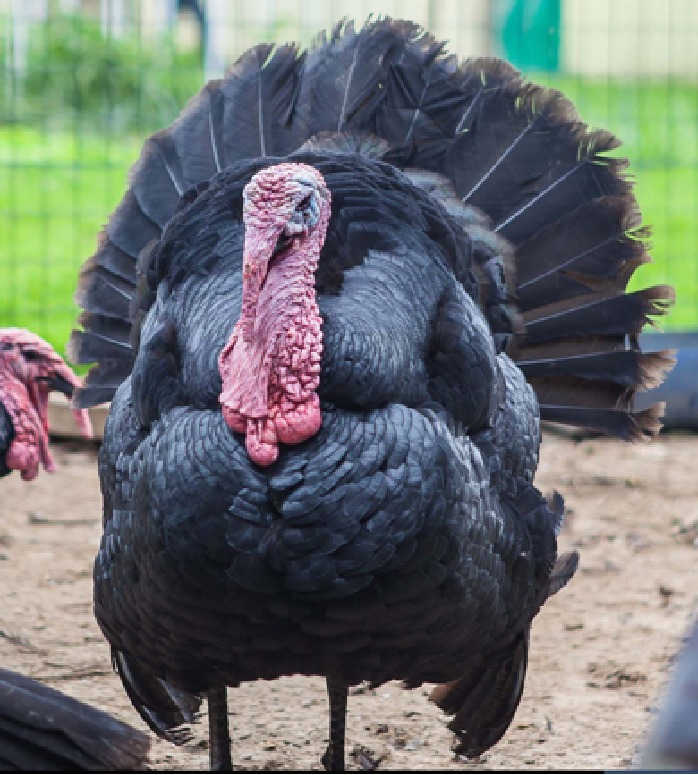 **Origin Area**: North Italy (Lombardia) **Weight (kg):** F:2.1-3-9; M: 4.9-7.1 **N. eggs/year:** 41 **Fertility:** 84-85% **Plumage:** Black. **Description:** Rustic breed with a strong hatching attitude Breeding in local areas.	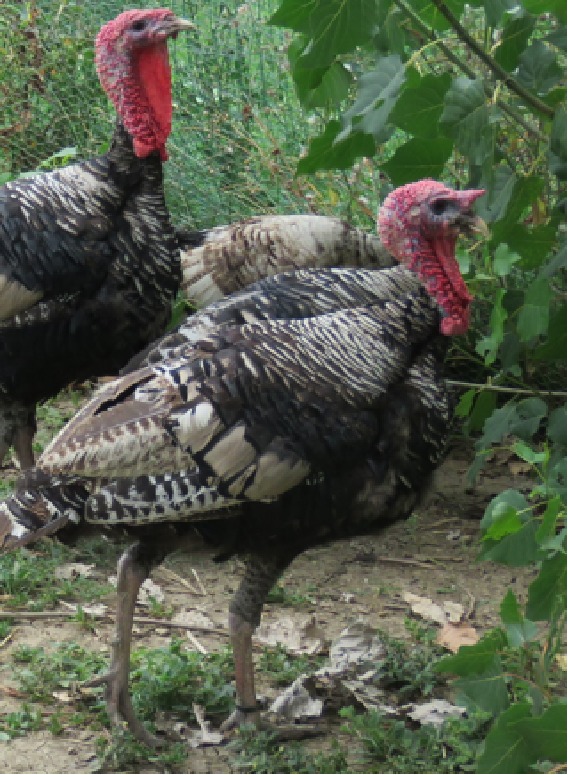 **Origin Area**: North Italy (Emilia Romagna) **Weight (kg):** F:6.5; M: 12 **N. eggs/year:** N/A **Fertility:** N/A **Plumage:** Steel gray with white streaks. **Description:** Local breeding, numerical consistency extremely small.	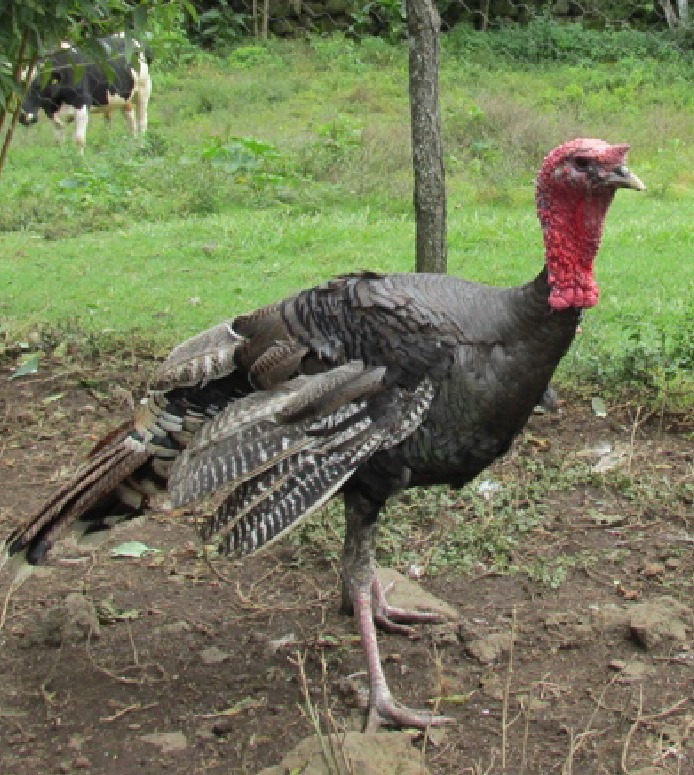 **Origin Area**: Mexico **Weight (kg):** F: 3.2; M: 5.7 kg **N. eggs/year:** N/A **Fertility:** N/A **Plumage:** Different colors. **Description:** Backyard birds. Unselected extremely variable in term of phenotype and production.	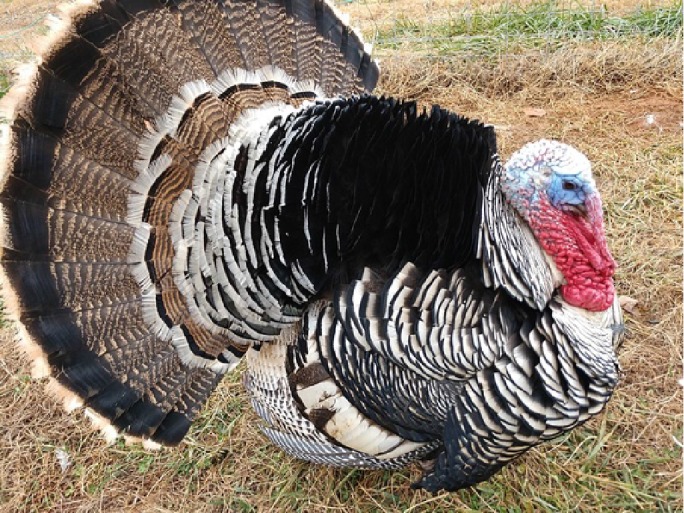 **Origin Area**: Rhode Island (USA) **Weight (kg):** F: 8.2; M: 15 kg **N. eggs/year:** N/A **Fertility:** N/A **Plumage:** Steel gray color. **Description:** Breeding in Europe and locally in Italy.

*Data from: https://www.pollitaliani.it/portfolio-articoli/razze/; **Data from: http://www.agraria.org/tacchini/neroitalia.htm; *** Data from: Utrera et al. (2016); §Picture from: https://commons.wikimedia.org/wiki/File:Narragansett_Turkey,_male.jpg

In the last 40 years the intensive selection in turkey produced a fast-growing meat bird, a commercial hybrid (HYB). The selection for heavy turkey started presumably in North America and preferred the white pigmentation to other plumage colors (Christman and Hawes, 1999; Ekarius, 2007). Birds are selected according to a strong directional mating system to improve weight at slaughter and feed conversion efficiency. The hybrid population here used is a common commercial line of selected heavy turkey (white plumage) that reaches a weight of 20 kg or more in males.

Even though the directional selection occurring in European populations for more than 500 years determined that breeds differentiated in morphology and in performances, the European and central American populations share a common genetic background, because their common ancestral origin. This holds true also for commercial turkey line where, nevertheless, the intense directional selection performed in the last 40 years, affected dramatically the physiology, the adult weight, the growth rate, the behavior and the bird’s sociality respect to the wild type^1^.

The Copy Number Variants (CNVs) are genomic structural variants recognized to have an active role in gene regulation (Redon et al., 2006; Gamazon and Stranger, 2015) and capable to identify genomic variation among populations. Their use in identifying genomic variation among populations is particularly relevant as several authors found a large proportion (up to 60% in chicken) of mapped CNVs Regions (CNVRs) harboring annotated genes related to expressed phenotypes caused by the specific evolution occurred in the populations (Gorla et al., 2017; Drobik-Czwarno et al., 2018; Strillacci et al., 2018).

The goal of this study is to produce the first CNV map in the Turkey species (*Meleagris gallopavo*) using high-density SNP chip information in several populations—the Mexican turkey, the Narragansett, six Italian breeds—and a commercial hybrid, and to produce a GO analysis of annotated genes in the mapped CNVRs. The strong directional selection occurring in high-producing hybrids, the one that occurred in the differentiation of the Narragansett and the Italian Turkey breeds, and the adaptive selection in the Mexican turkey population is then discussed according to the genes harbored in the CNVRs. The second goal of this study is to identify the existing variability among the breeds and populations using the mapped CNV, since knowledge of their genomic variation can be used to interpret the phenotypic variability.

## Materials and Methods

### Sampling and SNP Chip Processing

A total of 115 biological samples from individuals belonging to six Italian breeds (Colle Euganei: CoEu – 22; Bronzato Comune Italiano: BrCI – 5; Parma e Piacenza: PrPc – 15; Brianzolo: BR – 32; Nero d’Italia: NI – 31; Ermellinato di Rovigo: ErRo – 10), 7 Narragansett turkeys, 38 commercial hybrids (HYB), 30 Mexican turkeys (MEX) were available from previous collections or deriving from other research projects and part of the University of Milan repository of animal samples. The University of Milan permit for the use of collected samples in existing bio-banks was released with n. OPBA-56-2016. The Mexican sample collection is part of the institutional Project “Identificación de los recursos genéticos pecuarios para su evaluación, conservación y utilización sustentable en México. Aves y cerdos. SIGI NUMBER 10551832012” coordinated with the activities of the Centro Nacional of Recursos Genéticos (CNRG) at Tepatitlán, Jalisco (México)^2^. Original owners of sampled individuals gave consent for re-use for research purposes. The study did not require any ethical approval according to national rules, according to EU regulation, as it does not foresee sampling from live animals.

The samples of the Italian breeds belong to individuals originally collected in different areas of North Italy (Veneto, Lombardia and Emilia Romagna), in nine small farms dedicated to the breeding of one or two breeds each. The MEX individuals were originally sampled across 12 different states of Mexico, characterized by various climatic and geographical environments. The individuals belong to backyard small groups, spread over many small farms. These birds, to the best of our knowledge, did not undergo any selection by the owners, who let them reproduce according to a naturally occurring random mating as they are raised as a backyard population. The Narragansett individuals were originally sampled from two family farms in North Italy. A brief description of each turkey population including a picture, the sampling geographical area, the plumage color, the adult body weight, and the fertility performance are reported in **Table 1**. The commercial hybrid comes from a unique farm in the Lombardia region in north Italy from the same batch of birds.

DNA extraction from feathers (Mexican samples) and blood (all others) samples were performed using ZR Genomic DNA™ Tissue MiniPrep (Zymo, Irvine, CA, USA) according to the procedures relative to different tissues. DNA was quantified using NanoQuant Infinite^®^m200 (Tecan, Männedorf, Switzerland) and diluted to 50 ng/μl. Samples were processed on the Axiom^®^ Turkey Genotyping Array (Affimetrix) containing 634,067 SNPs. The Turkey_5.0 (GCA_000146605.1) genome assembly was used in this study as reference genome.

A quality control of raw intensity files using the standard protocol in the Affymetrix Power Tools package (www.affimetrix.com) was performed in order to guarantee high quality of obtained data. Default quality control settings, according to the manual (www.affimetrix.com), were applied to filter for low-quality samples, i.e. genotyping call rate <98% and Dish Quality Control <0.82.

### CNVs Detection and Subsequent Analysis

The Log R Ratio (LRR) and the B allele frequency (BAF) values were obtained using the Axiom^®^ CNV Summary Tool software. Outlier samples for LRR were identified using the SVS 8.4 software (Golden Helix Inc., Bozeman, MT, USA) through: i) the overall distribution of Derivative Log Ratio Spread (DLRS) values; and ii) screened according to GC content, which is correlated to a long-range waviness of LRR values by the wave detection factor algorithm as in Diskin et al. (2008).

The CNV detection was performed on the data of birds passing quality controls on 30 autosomes, using two different calling algorithms: i) the Copy Number Analysis Module of SVS^3^, and ii) the Hidden Markov Model of PennCNV software^4^. In order to reduce the false-positive calls a consensus map of CNV obtained by the two algorithms was produced.

The CNV calling performed with SVS has been obtained using the univariate analysis based on LRR values, with the following options: univariate outlier removal, a limit of not more than 100 segments per 10,000 markers with a minimum of three markers per segment, and 2,000 permutations per pair with a P-value cut off of 0.005, according to the SVS 8.4 user manual.

The PennCNV calling (Wang et al., 2007) was based on LRR and BAF values using the default parameters: standard deviation of LRR <0.30, BAF drift as 0.01 and waviness factor at 0.05, and a minimum of three SNPs required to define a CNV. In addition, as to reduce the false calling rate function of the hmm parameter file proper of PennCNV, the CNV call was obtained using three different “hmm” files (agre.hmm, affygw6.hmm, hh550.hmm). The online PennCNV manual describes that the agre.hmm file produces an excess of false-positive calls respect to the default affygw6.hmm file (both specific for Affymetrix SNP array), which instead is known to produce a low number of CNV calls (i.e. excess of false negative) with respect to other calling software and algorithms. The hh550.hmm file (specifically developed for Illumina SNP arrays) has been considered in the calling process, because is based on a SNPs chip density closest to the one used in this study.

After the four CNVs detections (i.e. one for each hmm file and the one from SVS8.4), the outputs were compared, at individual level and within each population, using the -intersectBed command of Bedtools software (Quinlan and Hall, 2010). For each individual, the consensus_CNVs were defined as the length of the DNA tract full overlapping across at least two detections. CNVs were classified in loss (0 and 1 from the PennCNV output) and in gain (3 and 4 from PennCNV output) and were constant across the different callings.

CNV regions (CNVRs) at population level were obtained by merging consensus_CNVs that overlap by at least 1 bp using the -megeBed command of Bedtools (Quinlan and Hall 2010) in at least two birds. The identified CNVRs were classified as “breed_CNVRs” and “shared_CNVRs”, when occurring in only one breed (i.e. BR, BrCl, CoEu, ErRo, NI and PrPc) or population (i.e. NARR MEX and HYB), or in at least two ones, respectively. CNVRs were classified within population in gain (all consensus_CNVs gain), loss (all consensus_CNV loss), and complex (consensus_CNVs both gain and loss). Singleton CNVs were considered also to be singleton CNVRs.

Genes were annotated within the CNVRs using the NCBI Turkey_5.0 gene dataset (annotation Release 102), and the Bedtools “-intersectBed” command was used to catalogue these genes to the corresponding regions. Gene Ontology terms (GO) and Kyoto Encyclopedia of Genes and Genomes (KEGG) pathway analyses were performed using the DAVID Bioinformatic Database^5^. Only LOC genes catalogued in NCBI Database as protein genes were considered.

Different approaches were used to disclose population structure and diversification of all turkey population. In order to provide the required input for different analyses two different matrices were built using CNV data: i) the first matrix (matrix_1) was built by assigning a value of “1” (presence of CNV), or “0” (normal state) to each sample-CNV for each CNVR, without considering the CNV state; ii) the second matrix (matrix_2) was built assigning the sample-CNV genotypes: “0” homozygous deletion, “1” heterozygous deletion, “2” normal state (absence of CNV in that region), “3” heterozygous duplication and “4” homozygous duplication. For details see Strillacci et al. (2018).

The Past software (Hammer et al., 2001) was employed to perform and visualize two principal component analyses (PCAs), the first based on the matrix_1 as input data, and the second based on matrix_2. In addition, two 3D PCAs were performed with the “rgl” package of R^6^ on PCAs results. The pvclust R package was utilized using the same matrixes to carry out two Hierarchical Clustering Analyses (HCA) applying 10,000 bootstraps (Suzuki and Shimodaira, 2006).

The STRUCTURE Software v.2.3.4 (Pritchard et al., 2000; Falush et al., 2003) was used to represent the population structure of the populations studied, on the basis of matrix_1. We used the STRUCTURE admixture model without the LocPrior option and setting 5,000 as burning period and 10,000 as iterations, performing five replicates for each K value from 2 to 20 and assuming nine different populations. Structure Harvester software (Dent and vonHoldt, 2012) was used to obtain the best K values, on the basis of STRUCTURE results, providing the DeltaK values according to the heuristic method reported by Evanno et al. (2005). The STRUCTURE PLOT software (Ramasamy et al., 2014) was employed to graphically visualize each cluster assignment of the K obtained.

## Results

### CNVs and CNVRs Maps

A total of 13 samples (5 NI, 2 PrPc, 4 MEX, 1 ErRo and 1 BR) were excluded during quality assurance: three because of high DLRS values, seven because of wave factor values, and three for their exceptionally high number of called CNVs. Consequently, the final CNV dataset was used for genomic variation analyses comprised a total of 177 turkeys.

**Figure 1:**
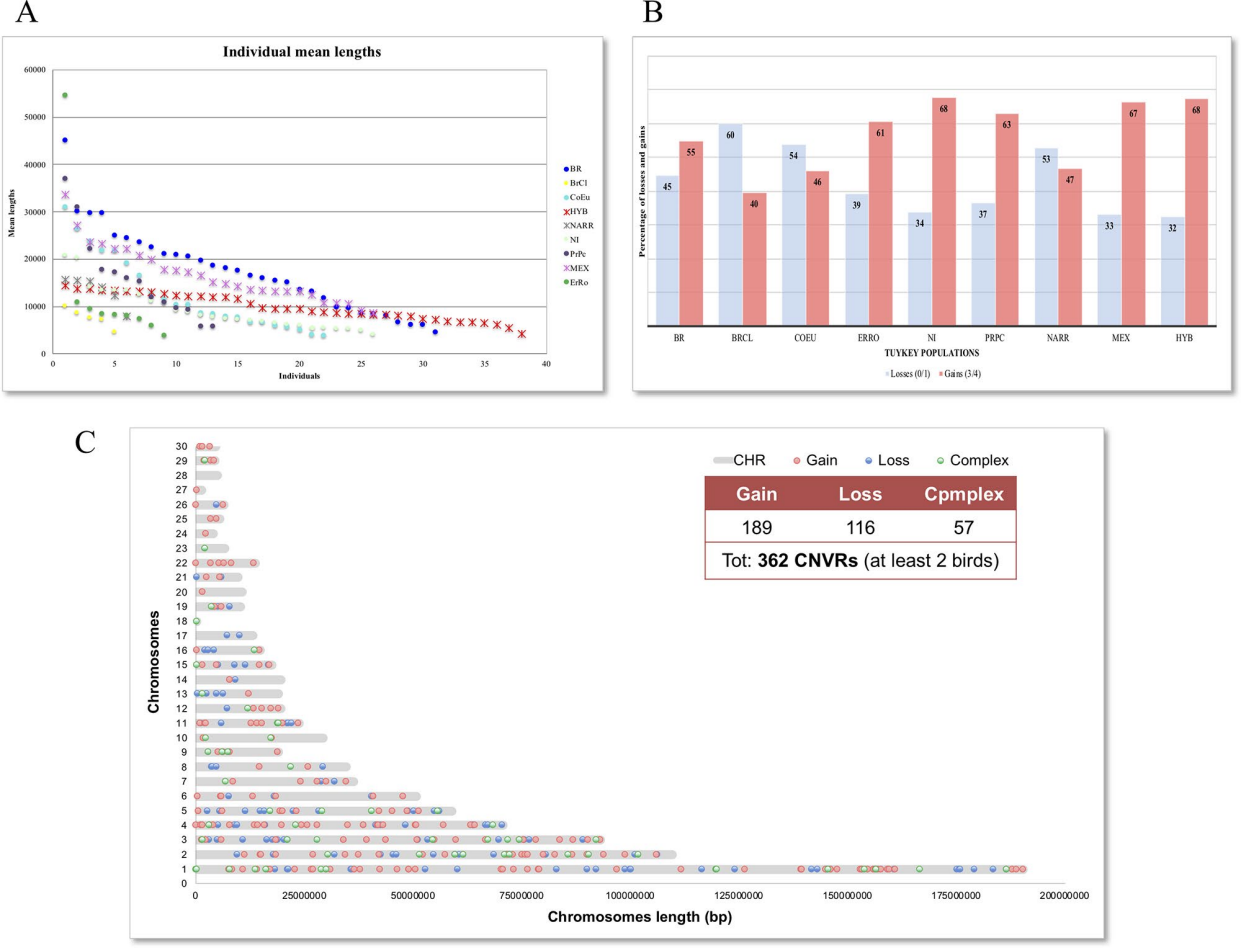
Graphical representation of identified CNVRs. **(A)** Distribution of Individual mean length for each population; **(B)** Percentage of losses and gains CNVRs in each population; **(C)** Map of CNVRs in the autosomes according with states.

The total number of CNVs called was 2,987 (**Supplementary Table 1**) and varied in terms of number and size among the individuals of each population, as reported in **Table 2**. CNVs ranged from 819 bp to 453.5 kb in size with an average length of 115.2 kb, covering a total length of about 41 Mb (4.65%) of the turkey genome (chromosomes 1–30). The BrCl and the HYB showed shorter average CNVs with respect to other populations, while in the MEX one the longest average CNVs length was found. The HYB birds are also the most homogeneous for the average length of CNVs (**Figure 1**). The MEX breed is the one with the largest number of CNVs per individual (i.e. 28) while the HYB is the one with the lowest (i.e. 10).

**Table 2:** Summary of CNVs identified in each population.

Breed	N. of samples	N. CNVs	CNV per sample Min-Max (average)	Loss (0/1)*	Gain (3/4)*	Min length	Max length	Mean length	Coverage	Total Coverage (%)
BR	31	412	4-34 (13)	185	227	1,221	214,517	15,715	6,474,485	0.73
BrCl	5	63	6-24 (12)	38	25	1,271	25,586	7,357	463,483	0.05
CoEu	22	354	8-37 (16)	191	163	1,096	184,966	11,762	4,163,692	0.47
ErRo	9	135	8-30 (10)	53	82	1,221	362,781	11,569	1,561,859	0.18
NI	26	567	6-69 (22)	192	375	1,096	283,259	12,436	7,038,934	0.8
PrPc	13	232	7-42 (18)	85	147	1,328	230,199	16,307	3,783,129	0.43
NARR	7	96	10-22 (14)	51	45	1,301	83,743	13,105	1,258,113	0.14
MEX	26	734	12-49 (28)	245	489	819	453,485	16,979	12,462,363	1.41
HYB	38	394	4-20 (10)	128	266	1,070	62,316	9,964	3,935,744	0.45
**Total**	**177**	**2,987**	**4-69 (17)**	**1,168**	**1,819**	**819**	**453,485**	**115,194**	**41,141,802**	**4.65**

*0 = homozygous deletion, 1 = heterozygous deletion, 3 = heterozygous duplication, and 4 = homozygous duplication.

Duplications were higher than deletions in the majority of populations except for BrCI, CoEu, and NARR breeds, where the ratio gain/loss (losses are the sum of the total copy numbers 0 and 1; gains are the sum of the total copy numbers 3 and 4) are inverted as shown in **Figure 1**. The gain/loss ratio is similar in HYB, MEX, NI, and PrPc populations (about 65% vs. 35%), but the proportion of duplication and deletion are differently represented in the other populations. The CNVRs including at least 2 individuals were 362 counting 189 gains, 116 losses, and 57 complexes and their distribution on the chromosomes is shown in **Figure 1**.

Statistics of CNVRs for each population are reported in **Table 3**. A total of 1,659 CNVRs (OverAll) were obtained across all populations with 412 Loss, 1,190 Gain, and 57 Complex.

**Table 3:** Summary of CNVRs identified for each turkey’s population.

Breed	CNVR	Loss	Gain	Complex	Min length	Max length	Mean length	Coverage	Total Coverage (%)
BR	223	53	168	2	1,221	214,517	12,293	2,741,386	0.31
BrCl	47	24	23	0	1,383	25,586	7,063	331,977	0.04
CoEu	195	56	138	1	1,096	186,030	10,542	2,055,612	0.23
ErRo	108	79	29	0	1,221	362,781	12,634	1,364,494	0.15
NI	358	58	293	7	1,096	283,259	15,186	5,436,564	0.62
PrPc	186	59	126	1	1,328	230,199	14,029	2,609,445	0.30
NARR	77	39	38	0	1,301	83,743	11,494	885,013	0.10
MEX	575	185	385	5	843	453,485	15,864	9,122,023	1.03
HYB	243	59	181	3	1,070	62,316	8,830	2,145,688	0.24
**OverAll**	**1,659**	**412**	**1190**	**57**	**843**	**453,485**	**13,612**	**22,581,871**	**2.55**

Details on CNVRs are reported in **Supplementary Table 2** for those including at least two individuals and detected across breeds, i.e. shared_CNVRs. The 1,297 singleton CNVRs, representing 64% of all detected ones are listed in the **Supplementary Table 3**. **Supplementary Figure 1** shows the distribution of singleton among breeds/populations and the distribution of loss and gain across all populations and by breed/population. The largest proportion of CNVRs resulted in gain, i.e. 77% across all breeds/populations, with a proportion of singletons of 64%. This result is consistent with the proportion of singleton identified in chickens by others (Yi et al., 2014; Gorla et al., 2017).

**Figure 2:**
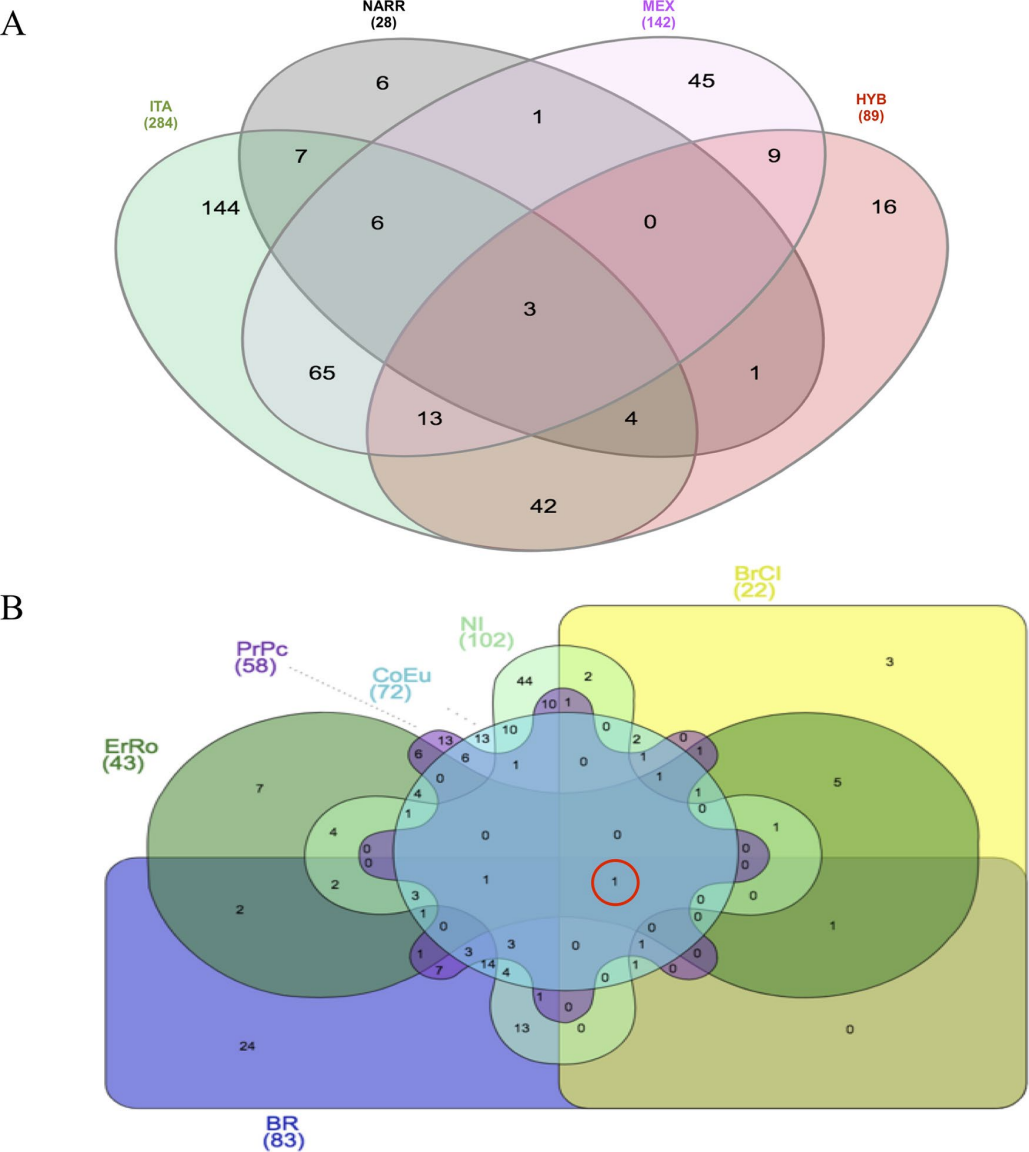
Venn diagrams of CNVRs identified: **(A)** in turkeys grouped according to ITA-breeds; NARR; MEX and HYB; **(B)** in the six Italian turkey breeds.

The Venn diagram (Heberle et al., 2015) shown in **Figure 2** represents the amount of CNVRs shared among the populations, grouping them as ITA (all Italian breeds), NARR (the Narragansett), MEX (the Mexican turkey population), and HYB (the commercial cross). The reason of this grouping resides in the type of evolution of the populations: the Italian breeds are all highly selected for breed standard phenotypes and possibly highly inbred; the Mexican population has been under an outbreeding mating system, with no directional selection undertaken for centuries; the NARR is a cross between the wild American turkey and the US domestic Bronze turkey; the HYB is a commercial population obtained by a strong directionally selection for heavy body weight. Three CNVRs resulted as common to all populations and a large proportion of ITA CNVRs are shared with MEX and HYB—65 and 42 CNVRs, respectively.

In **Table 4** the details of the 32 CNVRs detected in at least 10 samples and the genes lying in the same regions are reported. Among those, the three regions in common to all turkey populations, as shown also in **Figure 2**, are located on chr3 at 92,889,953–92,936,492 (CNVR_1126, gain), on chr4 at 26,993–164,704 (CNVR_1240, gain), and on chr4 at 68,446,449–68,522,752 (CNVR_1371, complex). In the CNVR_1371, the one also found in the largest number of individuals from all breeds, the *CD8A* gene that is related to immune and inflammatory response is annotated (Li et al., 1999). In the other two common regions, CNVR_1126 and the CNVR_1240, the *FK1L* and the *TLR2A* gene are annotated, respectively, and involved in immune and inflammatory response and in feather keratin multigene family with implication in feather evolution (Li et al., 2013; Velová et al., 2018).

**Table 4:** List of the CNVRs mapped in at least 10 birds with chromosome, start bp, end bp, CNVR length and CNVR state. For each of the CNVRs the count of birds for each population (ITA, NARR, MEX, HYB) is reported together with their total. The genes annotated in the each region are listed with the trait of interest and the reference.

N_CNVR	Chr	CNVR start	CNVR end	CNVR length	ITA						NARR	MEX	HYB	Total Samples	CNVR state	Genes	Trait by gene: (species)	References
					BR	BrCl	CoEu	ErRo	NI	PrPc								
CNVR_113	1	46402671	46430314	27643						1			17	18	gain			
CNVR_126	1	52847470	52853786	6316					9				4	13	loss			
CNVR_163	1	76320966	76430128	109162	6				8	7		2		23	gain	*OVSTL, TCRb1*	OVSTL: eggshell calcified layer (quail)	Mann and Mann, 2015
CNVR_206	1	98886764	98931838	45074						2			17	19	loss			
CNVR_210	1	99904908	99927304	22396	9		1			1			1	12	loss			
CNVR_307	1	145466178	145680695	214517	9				1				1	11	complex			
CNVR_757	2	30461083	30521978	60895			9					4		13	complex			
CNVR_780	2	42604981	42606860	1879	9	1		2				1		13	loss			
CNVR_809	2	57899261	57923296	24035					1		5	4		10	complex			
CNVR_843	2	72167387	72173022	5635	10		4		4					18	loss			
CNVR_920	2	101084671	101088748	4077		1		2	6		1		4	14	loss			
CNVR_1088	3	20396386	20399251	2865	10		18			11				39	loss			
CNVR_1152	3	54655570	54693060	37490					1			2	10	13	complex	*OPN5L1*		
CNVR_1226	3	92889953	92936492	46539	1	1	1			1	1	6	4	15	gain	*FK1L*		
CNVR_1240	4	26993	164704	137711	2		1		3	2	2	5	3	18	gain	*TLR2A*	host immune response (Birds)	Velová et al., 2018
CNVR_1243	4	1581791	1620844	39053						2			9	11	gain	*GRIA2*		
CNVR_1246	4	3011587	3071312	59725	5			2		1		4		12	complex	*FSTL5*		
CNVR_1259	4	8948522	8954649	6127	12				5					17	loss			
CNVR_1357	4	63830569	63837531	6962	21		19		24	1				65	loss			
CNVR_1358	4	63850913	63854111	3198	19		19		23	1				62	loss			
CNVR_1371	4	68446449	68522752	76303	4	4	13	7	13	2	2	7	16	68	complex	*CD8A*	host immune and inflammatory response (Poultry)	Yi et al., 2014
CNVR_1408	5	15840153	15842835	2682	3		10		1			4		18	loss			
CNVR_1586	7	28038559	28062433	23874	2		1	1	2	2		5	2	15	gain			
CNVR_1598	8	3846585	3850061	3476		1	8	1		2		2		14	loss	*PRKG1*	feeding efficiency (bovine)	Taye et al., 2017
CNVR_465	1.1.32 11	1.1.33 1004126	1.1.34 1053713	1.1.35 49587	1.1.36 1		2	1	2			6		12	gain	*HNRNPL*		
CNVR_488	11	18985991	19015763	29772	6		1	1	3		1			12	complex	*LMAN1, CPLX4*	LMAN1: feed efficiency and feeding behavior (pig)	Reyer et al., 2017
CNVR_644	16	4206442	4209316	2874	12		11					1		24	loss	*GRIN2A*		
CNVR_970	21	5878926	5903943	25017		5	3	2						10	loss			
CNVR_987	22	5386977	5429908	42931	2		1	1	2				4	10	gain	*SLC52A3, RSPO4, SRXN1*		
																*TCF15*	growth (bovine)	Paredes-Sánchez et al., 2015
																* FAM110A*	growth (human, bovine)	Espigolan et al. (2015)
																*ANGPT4*	birth weight (human)	Turan et al. (2012)
																*SCRT2*	self- reported helping behavior (human)	Primes and Fieder (2018)
CNVR_1003	24	2359444	2545474	186030	1		12							13	gain	*TACC1, PLEKHA2, TM2D2, ADAM9, IDO2, C24H8orf, ZMAT4*	(all genes) residual feed intake (bovine)	Hardie et al. (2017)
CNVR_1024	26	6388747	6431016	42269			1		2			19		22	gain			
CNVR_1025	27	157671	192870	35199			1		5			10		16	gain	*VPS45, NUPL2*		

The other two regions are shared by a large number of individuals of ITA breeds and have been detected on chr4 at 63,830,569–63,854,111 in CNVR_1357 (62 birds from ITA breeds) and CNVR_1358 (65 birds from ITA breeds). These two regions are both a loss, are very close on the genome being 13,382 bp apart, and have been detected in almost the same samples of the same ITA breeds. No genes are annotated within these two CNVRs. Ten CNVRs in **Table 4** are common to ITA and MEX, five common to ITA and HYB, and only one in common between ITA and NARR. Among these regions, nine of them include genes (CNVR_163, CNVR_1243, CNVR_1246, CNVR_1598, CNVR_488, CNVR_644, CNVR_987, CNVR_1025). There are no regions shared only among HYB, NARR, and MEX.

The Venn diagram in **Figure 2** shows in detail the distribution of CNVRs among the six Italian breeds. It is worthy to mention that the gene *CD8A* is in a CNVR common to all the Italian breeds (in the red circle).

Among the 362 CNVRs a total of 140 were mapped only in one specific population, the breed_CNVRs, as reported in **Supplementary Table 4**. The mapped genes in any species and the corresponding references for each association studies, the associated phenotypes and the organism involved, are also indicated.

The largest number of breed_CNVRs occurred in the MEX turkey population with 45 regions followed by the NI with 33. The lowest number of breed_CNVRs was found in the BrCI and in the NARR with one and four breed_CNVRs respectively. The number of genes annotated in the breed_CNVRs was 26 and 21 in MEX and NI, while the number of genes in breed_CNVR in other populations was between one and eight. The gene *IMMPL2* is harbored by two breed_CNVRs, one in the BI (CNVR_69) and one in the NI (CNVR_70). The two regions are very close even if they do not overlap.

The results of the GO TERM and KEGG pathway analyses obtained using DAVID considering the genes found in the 362 shared_CNVRs are reported in the **Supplementary Table 5** into clustered and not clustered groups of genes.

**Supplementary Table 6** contains the information generated from the KEGG and GO Term analyses using DAVID from breed_CNVRs. The information was obtained using *Meleagris gallopavo* as background species and integrated and confirmed using the *Gallus gallus* as background, in case of absence of complete information for the *Meleagris gallopavo* species.

### Genetic Variability Across Turkey Populations

**Figure 3:**
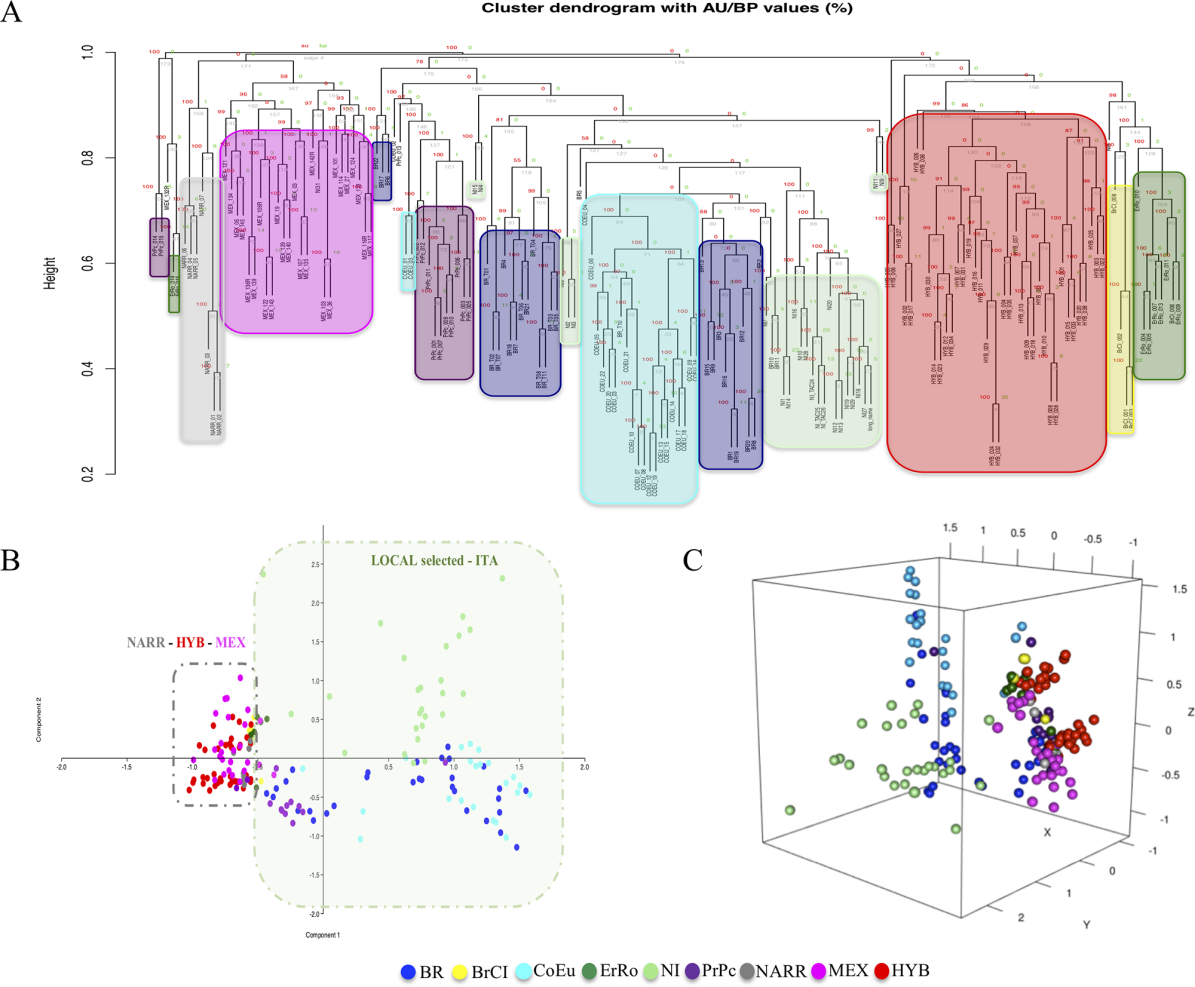
Hierarchical clustering and PCAs based on CNVRs (CNV encoded as in matrix_1). Panels **(A)**, **(B)**, and **(C)** are the dendrogram, the PCA-2D, and the PCA-3D, respectively.

**Figure 4:**
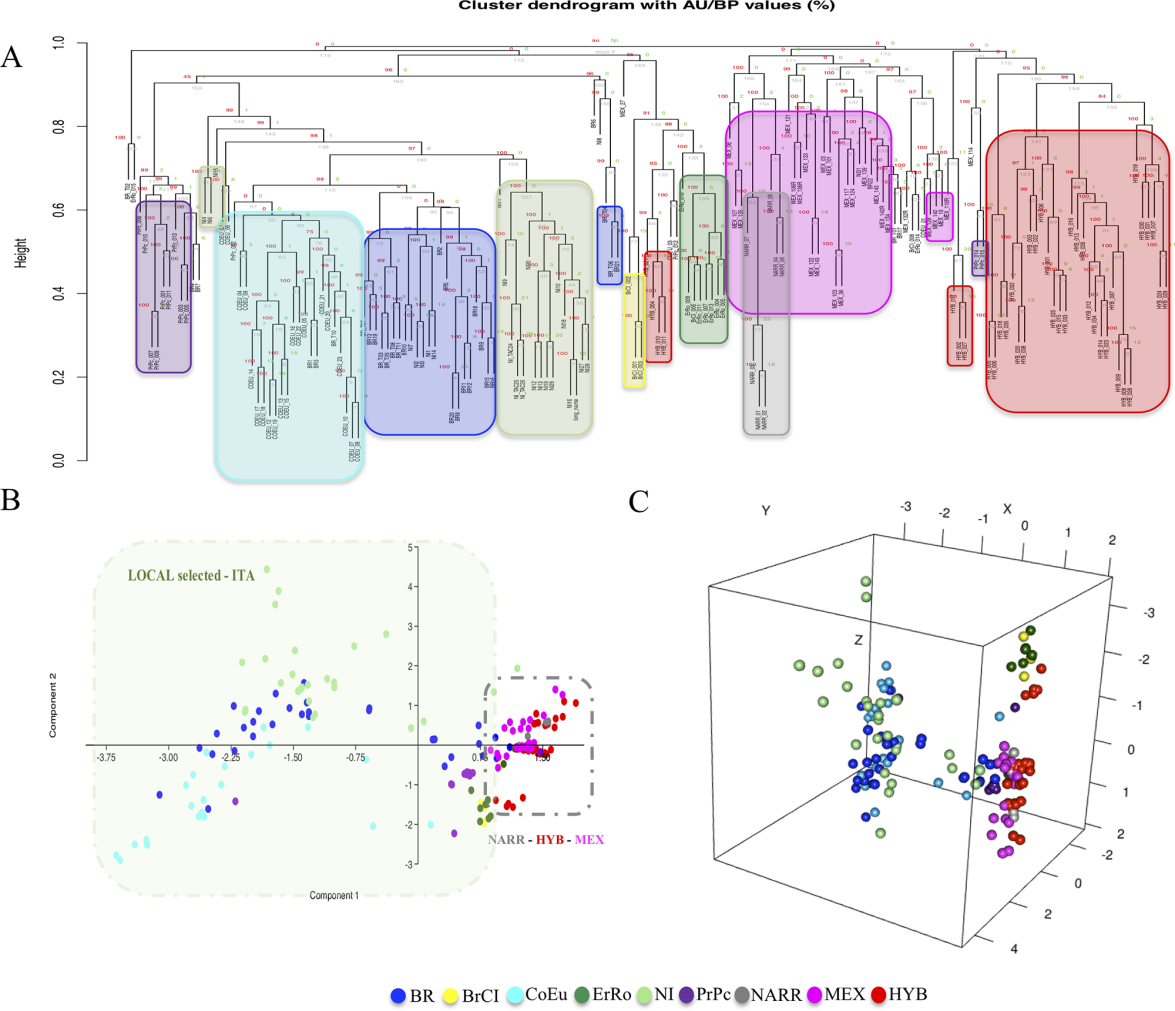
Hierarchical clustering and PCAs based on CNVRs (CNV encoded as in matrix_2). Panels **(A)**, (**B)**, and **(C)** are the dendrogram, the PCA-2D, and the PCA-3D, respectively.

Two clustering analysis were performed based on two different matrices (matrix_1 and matrix_2) described herein before. Both the cluster dendrograms, **Figure 3** based on matrix_1 and **Figure 4** based on matrix_2, showed distinct clades grouping animals belonging to the same populations. It is interesting to note that MEX and NARR always clustered very close. Also, Italian breeds and the Hybrid group form well distinct clusters according to their origin.

In all the PCAs graphs in **Figures 3B, C** and **4B, C** the clustering results show two main clades: NARR, MEX, and HYB were grouping closer, while the ITA breeds clustered in a separate one.

The STRUCTURE software was employed to infer population structure and gene flow of the individuals of the nine populations studied. We calculate a number of K from K = 2 to K = 20 to identify the true number of possible clusters (subpopulation) in which it is possible to divide the populations. The estimated likelihood [LnP (D)] values were used to find the ΔK to distinguish the break in slope of the distribution of LnP (D) values at the true K. The analyses identify K = 13 as the best likely K value, suggesting that the population could be divided into 13 genetic groups.

Even though K = 5 shows the second higher value (**Supplementary Figure 2**) it is not possible to well differentiate the populations as in K = 13. In fact, for K = 2 to K = 12 it is not possible to assign each population to a clear distinct cluster, while for K = 14 to K = 20 the high level of admixture in each of the population results in nonsignificant successive clustering.

## Discussions

The results from this study are likely reflecting the human action on turkey populations, i.e., its migration to Europe and then back to America, and the directional selection occurring in the last 40 years to produce a fast-growing heavy bird.

The study considers three main groups of birds that reproduce and adapt according to different constraints and environmental conditions. The MEX population developed in a natural environment, with no (or very little) intervention by humans in mating and with no (or very little) supplement of feed and harsh rearing conditions. The Italian populations are the result of a phenotypic selection operated by individual farmers in their small group of individuals and operated to obtain birds that best perform in the semi-extensive farming system (backyard with recovery availability and feeding supplement) that characterized the middle ages poultry system of Italy and Europe. The HYB population, in the last 40 years, has been heavily directionally selected, through very well-structured genetic improvement and breeding plans to improve weight and growing performances and to best perform in an artificially controlled environment with unlimited feed supplement.

Our study is the first CNV mapping in a worldwide turkey sampling, from populations collected across different continents, and disclosed similarities and variation in CNVs and CNVRs across the populations studied. Because of the diversity in their breeding history and actual farming environmental conditions the MEX, ITA, and HYB populations provide an interesting model to investigate CNV variation, and their relation to gene expression and rearing conditions. The CNVs, in fact, are widely recognized to be a non-neutral genomic structural variation related to positive and directional selection. The CNV has been recently successfully used in poultry to differentiate breeds and populations with different genetic background (Gorla et al., 2017; Strillacci et al., 2017; Sohrabi et al, 2018), as well as in other species (Xu et al., 2016; Strillacci et al., 2018). Interestingly in chicken Sohrabi et al. (2018) discuss long-term adaptation of animals to rural and hard rearing conditions in relation to a specific expressed trait linked to a CNV identified in the Creeper indigenous chicken local population that is adapted to the harsh environmental condition of southeastern Iran. Additionally, a recent study on a eukaryotic model (Hull et al., 2017) showed that environmental changes are accelerating adaptation through the stimulation of copy number variation and that this is not a random effect but has a cause-effect relationship. Perry et al. (2007) also demonstrated that directional selection due to starch diet (i.e. environmental factor) is increasing specific copies of the genes involved in starch metabolism producing as such CNV gains. The CNV difference among populations here is shown in particular by the variation in the number of CNV per bird that is the lowest in the HYB (10 on average) and the largest in the MEX (28 CNV) and by the CNV length that in the HYB is much less variable than in the other two group (ITA and MEX) of birds. These findings support the hypothesis that the variability in CNV (size and number), as in the MEX vs. the HYB, is possibly related to the different breeding and selection underwent in these populations and to the environmental conditions where they were farmed: MEX very harsh rearing, HYB controlled artificial environment and *ad libitum* feeding. The same holds true for the ITA vs. MEX and HYB.

Most of the genes found do not show previous associations with any specific function or pathway in turkey, since association studies in turkey are only few, but most of those genes have been previously studied and linked to functions in other species such as chicken, pig, bovine, birds, mice, zebrafish and human, as reported in **Supplementary Table 4**.

Thirty-two **r**egions were detected in at least 10 individuals, and 14 of them include 29 genes that are known to be involved in different traits in different species (**Table 4**), such as immune response (*TLR2A and CD8A),* feather evolution (*FK1L*), feed efficiency (*PRKG1* and *LMAN1),* growth traits (*TCF15, FAM110A*), and residual feed intake (*TACC1, PLEKHA2, TM2D2, ADAM9, IDO2, C24H8orf4, ZMAT4*), as reported in **Table 4**.

There are three CNVRs in common among all the populations; one of them harbors the *CD8A* gene, which is known to have a role in the host immune and inflammatory response in chicken (Li et al., 1999). The polymorphism of the *CD8A* gene has been studied in five lines of turkey populations by Li et al. (1999) who found a loss of this gene in one half of the turkey of a studied line. This loss can be related to the CNVR_1371 found in this study where 34 CNVRs were loss and 34, gain. All the ErRo resulted to have a loss—CoEu had 12 loss (over 13 birds), BrCI 4 loss (over five birds), while other populations have a more balanced representation between loss and gain CNVs.

The *TLR2A* gene has been shown to be involved in the bird’s evolution with a strong driving of TLR due to positive selection (Velová et al., 2018). It is interesting to note that our results show that CNVR_1240 include the *TLR2A* gene with only normal and gain state. Even if the question of the adaptive value of the TLR genetic variation is still unresolved, the results found here are supporting the hypothesis that positive selection is driving the evolution of the gene towards duplication of copies as proposed recently by Velová et al. (2018).

Other genes in the CNVRs found here (**Supplementary Table 4**) are associated with immunity and inflammatory response in mice (*TCF7*, *ARHGEF5*), chicken (*VMO1, GUCY1A2, NBN*), bovine (*NEK11*), and in all species (*PARP15*) as reported in previous studies (Wang et al., 2009; Daugherty et al., 2014; Strillacci et al., 2014; Jang et al., 2015; Lim and Song 2015; Zhu et al., 2015; Saelao et al., 2018; Velová et al., 2018). Among the genes reported in the **Supplementary Table 4**, the *IMMP2L* gene lies in CNVR_69 which is common to NI and BR. This gene was associated with fertility in mice (Bharadwaj et al., 2014) and with collective behavior in zebrafish (Tang et al., 2018). The presence of this gene in a gain CNVR may have some link with the typical collective behavior of the turkey.

## Conclusions

This study represents the first CNV mapping using high-density SNP chip on turkey. It provides first insights into the genomic architecture of the turkey population, laying the groundwork for future structural variation investigation in turkey species. In this study we have focused on the CNV, a structural variation linked to phenotypic expression regulation, in order to identify similarities across populations of the structural genome covered by this large variation.

The turkey populations are a unique resource to identify evolutionary processes affecting the structural genome since it is possible to access to populations under positive selection only and, on the other extreme, under heavy artificial selection. The most complete isolation of the MEX turkey population and the European ones together to the HYB provide a unique model to disclose the effect of the adaptation to environment and directional artificial selection performed by humans on the structural genome.

## Data Availability Statement

The raw data supporting the conclusions of this manuscript are included in the **Supplementary Materials**.

## Ethics Statement

The animal study was reviewed and approved by Università degli Studi di Milano - OPBA-56-216.

## Author Contributions

MS, AB, and SR-P conceptualized and designed the work. SC, SR-P, MS, AR-U, and MM-B collected the sample and data. MS and EG analyzed and interpreted the data. MS and AB wrote the manuscript. AG-R, MM-B, SR-P and VV-M made a critical revision of the manuscript. All authors have approved the final version of manuscript to be published.

## Funding

This research was co-funded by projects M01678 and 2016-PGR00206 - Ministries of Foreign Affairs of Italy and Mexico. Internal funding of the Università degli Studi di Milano

## Conflict of Interest

The authors declare that the research was conducted in the absence of any commercial or financial relationships that could be construed as a potential conflict of interest.

## Footnotes

1 https://www.coe.int/t/e/legal_affairs/legal_co-operation/biological_safety_and_use_of_animals/farming/Rec%20Turkeys.asp

2 http://www.inifap.gob.mx/SitePages/centros/cnrg.aspx

3 3http://goldenhelix.com

4 http://penncnv.openbioinformatics.org/en/latest/

5 https://david.ncifcrf.gov/tools.jsp

6 https://CRAN.R-project.org/package=rgl

## Supplementary Material

Supplementary Figure S1 Statistics of Singleton regions **(A)** Singleton % according to populations; **(B)** Singleton % according to state; **(C)** Graphical representation of Singleton % according to state and populations.

Supplementary Figure S2
**(A)** Plot representing the number of K groups that best fit the data; **(B)** Structure plot representing the degree of admixture among all the individuals of the nine populations considered for the two best K (K = 13 and K = 5).

Supplementary Table S1 CNVs identified in each Turkey population.

Supplementary Table S2 CNVRs with at least 2 individuals and detected in more than one turkey population.

Supplementary Table S3 Singleton CNVRs.

Supplementary Table S4 CNVRs with at least 2 individuals and detected only in one population (breed_CNVRs) (sheet1); References related to sheet1 (sheet2).

Supplementary Table S5 Gene annotation performed using DAVID Database. Sheet1 = clustered genes; Sheet2: non-clustered genes.

Supplementary Table S6 Annotation of all genes mapping within the breed_CNVRs.

## References

[B1] Bharadwaj M. S. Zhou Y. Molina A. J. Criswell T. Lu B. (2014). Examination of bioenergetic function in the inner mitochondrial membrane peptidase 2-like (Immp2l) mutant mice. Redox Biol. 2, 1008–1015. 10.1016/j.redox.2014.08.006 25460737 PMC4215389

[B2] Boije H. Harun-Or-Rashid M. Lee Y. J. Imsland F. Bruneau N. Vieaud A. (2012). Sonic Hedgehog-signalling patterns the developing chicken comb as revealed by exploration of the pea-comb mutation. PLoS One. 7 (12), e50890. 10.1371/journal.pone.0050890 23227218 PMC3515514

[B3] Cavalchini L. G. (1983). IL TACCHINO allevamento, incubazione, patologia Vol. 1 Edition. Edagricole, Italy. 304 pages.

[B4] Christman C. J. Hawes R. O. (1999). Birds of a Feather: Saving Rare Turkeys from Extinction. ALBC, Pittsboro, NC, USA.

[B5] Condro M. C. White S. A. (2014). Recent advances in the genetics of vocal learning. Comp. Cogn. Behav. Rev. 9, 75–98. 10.3819/ccbr.2014.90003 26052371 PMC4457475

[B6] Crawford R. D. (1992). Introduction to Europe and diffusion of domesticated turkey from the America. Arch. Zootec. 41, 307–314. 10.1007/978-1-4615-3426-6_27

[B7] Daugherty M. D. Young J. M. Kerns J. A. Malik H. S. (2014). Rapid evolution of PARP genes suggests a broad role for ADP-ribosylation in host-virus conflicts. PLoS Genet. 10 (5), e1004403. 10.1371/journal.pgen.1004403 24875882 PMC4038475

[B8] Day A. E. Quilter C. R. Sargent C. A. Mileham A. J. (2002). Characterization of the porcine sperm adhesion molecule gene SPAM1–expression analysis, genomic structure, and chromosomal mapping. Anim Genet. 33 (3), 211–214. 10.1046/j.1365-2052.2002.00830.x 12030925

[B9] Dent E. A. von Holdt B. M. (2012). STRUCTURE HARVESTER: a website and program for visualizing STRUCTURE output and implementing the Evanno method. Cons. Genet. Res. 4 (2), 359–361. 10.1007/s12686-011-9548-7

[B10] Diskin S. J. Li M. Hou C. Yang S. Glessner J. Hakonarson H. (2008). Adjustment of genomic waves in signal intensities from whole-genome SNP genotyping platforms. Nucleic. Acids. Res. 36, e126. 10.1093/nar/gkn556 18784189 PMC2577347

[B11] Drobik-Czwarno W. Wolc A. Fulton J. Dekkers J. C. (2018). Detection of copy number variations in brown and white layers based on genotyping panels with different densities. Genet. Sel. Evol. 50 (1), 54. 10.1186/s12711-018-0428-4 30400769 PMC6219011

[B12] Ekarius C. (2007). “Storey's Illustrated Guide to Poultry Breeds,” in Carol Ekarius, vol. 1 edition. (LLC: Storey Publishing).

[B13] Espigolan R. Baldi F. Boligon A. A. Souza F. R. Fernandes Júnior G. A. Gordo D. G. (2015). Associations between single nucleotide polymorphisms and carcass traits in Nellore cattle using high-density panels. Genet. Mol. Res. 14 (3), 11133–11144. 10.4238/2015.September.22.7 26400344

[B14] Evanno G. Regnaut S. Goudet J. (2005). Detecting the number of clusters of individuals using the software STRUCTURE: A simulation study. Mol. Ecol. 14 (8), 2611–2620. 10.1111/j.1365-294X.2005.02553.x 15969739

[B15] Fang M. Du H. Hu Y. Zhou X. Ouyang H. Zhang W. (2011). Identification and characterization of the pig ABIN-1 gene and investigation of its association with reproduction traits. J. Genet. 90 (1), e10–e20. 10.1007/s12041-011-0025-6 21677409

[B16] Falush D. Stephens M. Pritchard J. K. (2003). Inference of population structure using multilocus genotype data: linked loci and correlated allele frequencies. Genetics 164 (4), 1567–1587 12930761 10.1093/genetics/164.4.1567 PMC1462648

[B17] Gorla E. Cozzi M. C. Román-Ponce S. I. Ruiz López F. J. Vega-Murillo V. E. Cerolini S. (2017). Genomic variability in Mexican chicken population using copy number variants. BMC Genetics 18 (1), 61. 10.1186/s12863-017-0524-4 28673234 PMC5496433

[B18] Gamazon E. R. Stranger B. E. (2015). The impact of human copy number variation on gene expression. Brief. Funct. Genomics 14 (5), 352–357. 10.1093/bfgp/elv017 25922366 PMC4592354

[B19] Hammer Ø. Harper D. A. T. Ryan P. D. (2001). PAST: Paleontological statistics software package for education and data analysis. Palaeontol. Electronica 4 (1), 9.

[B20] Hardie L. C. VandeHaar M. J. Tempelman R. J. Weigel K. A. Armentano L. E. Wiggans G. R. (2017). The genetic and biological basis of feed efficiency in mid-lactation Holstein dairy cows. J. Dairy. Sci. 100 (11), 9061–9075. 10.3168/jds.2017-12604 28843688

[B21] Heberle H. Meirelles G. V. da Silva F. R. Telles G. P. Minghim R. (2015). InteractiVenn: a web-based tool for the analysis of sets through Venn diagrams. BMC Bioinf. 16, 169. 10.1186/s12859-015-0611-3 PMC4455604 25994840

[B22] Hull R. M. Cruz C. Jack C. V. Houseley. J. (2017). Environmental change drives accelerated adaptation through stimulated copy number variation. PLoS Biol. 15 (6), e2001333. 10.1371/journal.pbio.2001333 28654659 PMC5486974

[B23] Jang H. J. Lee H. J. Kang K. S. Song K. D. Kim T. H. Song C. S. (2015). Molecular responses to the influenza A virus in chicken trachea-derived cells. Poult. Sci. 94 (6), 1190–1201. 10.3382/ps/pev033 25877411

[B24] Letunic I. Bprk P. (2016). Interactive tree of life (iTOL) v3: an online tool for the display and annotation of phylogenetic and other trees. Nucleic Acids Res. 44 (W1), W242–W245. 10.1093/nar/gkw290 27095192 PMC4987883

[B25] Li Y. I. Kong L. Ponting C. P. Haerty W. (2013). Rapid evolution of Beta-keratin genes contribute to phenotypic differences that distinguish turtles and birds from other reptiles. Genome Biol. Evol. 5 (5), 923–933. 10.1093/gbe/evt060 23576313 PMC3673632

[B26] Li Z. Nestor K. E. Saif Y. M. Fan Z. Luhtala M. Vainio O. (1999). Cross-reactive anti-chicken CD4 and CD8 monoclonal antibodies suggest polymorphism of the turkey CD8alpha molecule. Poult. Sci. 78 (11), 1526–1531. 10.1093/ps/78.11.1526 10560824

[B27] Lim W. Song G. (2015). Differential expression of vitelline membrane outer layer protein 1: hormonal regulation of expression in the oviduct and in ovarian carcinomas from laying hens. Mol. Cell Endocrinol. 399, 250–258. 10.1016/j.mce.2014.10.015 25458700

[B28] Lovell P. V. Carleton J. B. Mello C. V. (2013). Genomics analysis of potassium channel genes in songbirds reveals molecular specializations of brain circuits for the maintenance and production of learned vocalizations. BMC Genomics 14, 470. 10.1186/1471-2164-14-47. 23845108 PMC3711925

[B29] Mann K. Mann M. (2015). Proteomic analysis of quail calcified eggshell matrix: a comparison to chicken and turkey eggshell proteomes. Proteome Sci. 13, 22. 10.1186/s12953-015-0078-1 26312056 PMC4550075

[B30] Paredes-Sánchez F. A. Sifuentes-Rincón A. M. Cabrera A. S. Pérez C. A. G. Bracamonte G. M. P. Morales P. A. (2015). Associations of SNPs located at candidate genes to bovine growth traits, prioritized with an interaction networks construction approach. BMC Genetics 16, 91. 10.1186/s12863-015-0247-3 26198337 PMC4511253

[B31] Pelz L. Purfürst B. Rathjen F. G. (2017). The cell adhesion molecule BT-IgSF is essential for a functional blood–testis barrier and male fertility in mice. J. Biol. Chem. 292 (52), 21490–21503. 10.1074/jbc.RA117.000113 29123028 PMC5766970

[B32] Perry G. H. Dominy N. J. Claw K. G. Lee A. S. Fiegler H. Redon R. (2007). Diet and the evolution of human amylase gene copy number variation. Nat. Genet. 39 (10), 1256–1260. 10.1038/ng2123 17828263 PMC2377015

[B33] Pickrell J. K. Pritchard J. K. (2012). Inference of population splits and mixtures from genome-wide allele frequency data. PLoS Genet. 8 (11), e1002967. 10.1371/journal.pgen.1002967 23166502 PMC3499260

[B34] Pinto D. Darvishi K. Shi X. Rajan D. Rigler D. Fitzgerald T. (2011). Comprehensive assessment of array-based platforms and calling algorithms for detection of copy number variants. Nat Biotechnol. 29 (6), 512–520. 10.1038/nbt.1852 21552272 PMC3270583

[B35] Primes G. Fieder M. (2018). Real-life helping behaviours in North America: a genome-wide association approach. PLoS One. 13 (1), e0190950. 10.1371/journal.pone.0190950 29324852 PMC5764334

[B36] Pritchard J. K. Stephens M. Donnelly P. (2000). Inference of population structure using multilocus genotype data. Genetics. 155 (2), 945–959. 10835412 10.1093/genetics/155.2.945 PMC1461096

[B37] Ramasamy R. K. Ramasamy S. Bindroo B. B. Naik V. G. (2014). STRUCTURE PLOT: a program for drawing elegant STRUCTURE bar plots in user friendly interface Vol. 3 Springerplus, 431. 10.1186/2193-1801-3-431. PMC4141070 25152854

[B38] Redon R. Ishikawa S. Fitch K. R. Feuk L. Perry G. H. Andrews T. D. (2006). Global variation in copy number in the human genome. Nature. 444 (7118), 444–454. 10.1038/nature05329 17122850 PMC2669898

[B39] Reich D. Thangaraj K. Patterson N. Price A. L. Singh L. (2009). Reconstructing Indian population history. Nature. 461 (7263), 489–494. 10.1038/nature08365 19779445 PMC2842210

[B40] Reyer H. Shirali M. Ponsuksili S. Murani E. Varley P. F. Jensen J. (2017). Exploring the genetics of feed efficiency and feeding behaviour traits in a pig line highly selected for performance characteristics. Mol. Genet. Genomics. 292 (5), 1001–1011. 10.1007/s00438-017-1325-1 28500374 PMC5594041

[B41] Quinlan A. R. Hall I. M. (2010). BEDTools: a flexible suite of utilities for comparing genomic features. Bioinformatics. 26 (6), 841–842. 10.1093/bioinformatics/btq033 20110278 PMC2832824

[B42] Saelao P. Wang Y. Gallardo R. A. Lamont S. J. Dekkers J. M. Kelly T. (2018). Novel insights into the host immune response of chicken Harderian gland tissue during Newcastle disease virus infection and heat treatment. BMC Vet. Res. 14 (1), 280. 10.1186/s12917-018-1583-0 30208883 PMC6134752

[B43] Schorger A. W. (1996). The Wild Turkey. Its History and Domestication. University of Oklahoma Press, Norman.

[B44] Strillacci M. G. Cozzi M. C. Gorla E. Mosca F. Schiavini F. Román-Ponce S. I. (2017). Genomic and genetic variability of six chicken populations using single nucleotide polymorphism and copy number variants as markers. Animal. 11 (5), 737–745. 10.1017/S1751731116002135. 27819220

[B45] Strillacci M. G. Frigo E. Schiavini F. Samoré A. B. Canavesi F. Vevey M. (2014). Genome-wide association study for somatic cell score in Valdostana Red Pied cattle breed using pooled DNA. BMC Genetics 15, 106. 10.1186/s12863-014-0106-7 25288516 PMC4198737

[B46] Strillacci M. G. Gorla E. Cozzi M. C. Vevey M. Genova F. Scienski K. (2018). A copy number variant scan in the autochthonous Valdostana Red Pied cattle breed and comparison with specialized dairy populations. PLoS One 13 (9), e0204669. 10.1371/journal.pone.0204669. 30261013 PMC6160104

[B47] Suzuki R. Shimodaira H. (2006). Pvclust: an R package for assessing the uncertainty in hierarchical clustering. Bioinformatics. 22 (12), 1540–1542. 10.1093/bioinformatics/btl117 16595560

[B48] Sohrabi S. S. Mohammadabadi M. Wu D. D. Esmailizadeh A. (2018). Detection of breed-specific copy number variations in domestic chicken genome. Genome. 61 (1), 7–14. 10.1139/gen-2017-0016. 28961404

[B49] Tang W. Zhang G. Serluca F. Li J. Xiong X. Coble M. (2018). Genetic architecture of collective behaviors in zebrafish. bioRxiv. 1, 350314. 10.1101/350314

[B50] Tardif S. Akrofi A. S. Dass B. Hardy D. M. MacDonald C. C. (2010). Infertility with impaired zona pellucida adhesion of spermatozoa from mice lacking TauCstF-64. Biol Reprod. 83 (3), 464–472. 10.1095/biolreprod.109.083238 20463354 PMC2924806

[B51] Taye M. Kim J. Yoon S. H. Lee W. Hanotte O. Dessie T. (2017). Whole genome scan reveals the genetic signature of African Ankole cattle breed and potential for higher quality beef. BMC Genetics 18 (1), 11. 10.1186/s12863-016-0467-1 28183280 PMC5301378

[B52] Turan N. Ghalwash M. F. Katari S. Coutifaris C. Obradovic Z. Sapienza C. (2012). DNA methylation differences at growth related genes correlate with birth weight: a molecular signature linked to developmental origins of adult disease. BMC Med. Genomics 5, 10. 10.1186/1755-8794-5-10 22498030 PMC3359247

[B53] Utrera A. R. Ponce S. I. R. Izquierdo A. V. Torres E. C. Covarrubias A. C. La Cruz Colín L. (2016). Analysis of morphological variables in Mexican backyard turkeys (Meleagris gallopavo gallopavo). Rev. Mex. Cienc. Pecuarias. 7 (3), 377–389.

[B54] Velová H. Gutowska-Ding M. W. Burt D. W. Vinkler M. (2018). Toll-like receptor evolution in birds: gene duplication, pseudogenization, and diversifying selection. Mol. Biol. Evol. 35 (9), 2170–2184. 10.1093/molbev/msy119 PMC6107061 29893911

[B55] Wang K. Li M. Hadley D. Liu R. Glessner J. Grant S. F. (2007). PennCNV: an integrated hidden Markov model designed for high-resolution copy number variation detection in whole-genome SNP genotyping data. Genome Res. 17 (11), 1665–1674. 10.1101/gr.6861907 17921354 PMC2045149

[B56] Wang Z. Kumamoto Y. Wang P. Gan X. Lehmann D. Smrcka A. V. (2009). Regulation of immature dendritic cell migration by RhoA guanine nucleotide exchange factor Arhgef5. J. Biol. Chem. 284 (42), 28599–28606. 10.1074/jbc.M109.047282 19713215 PMC2781403

[B57] Xu L. Hou Y. Bickhart D. M. Yang Z. Hay el H. A. Song J. (2016). Population-genetic properties of differentiated copy number variations in cattle. Sci. Rep. 6, 23161. 10.1038/srep23161 27005566 PMC4804293

[B58] Yi G. Qu L. Liu J. Yan Y. Xu G. Yang N. (2014). Genome-wide patterns of copy number variation in the diversified chicken genomes using next-generation sequencing. BMC Genomics 15, 962. 10.1186/1471-2164-15-962 25378104 PMC4239369

[B59] Zhu Y. Wang W. Wang X. (2015). Roles of transcriptional factor 7 in production of inflammatory factors for lung diseases. J. Transl. Med. 13, 273. 10.1186/s12967-015-0617-7 26289446 PMC4543455

