# Supplementary figures and images for: Copy Number Variation Mapping and Genomic Variation of Autochthonous and Commercial Turkey Populations
Source: Front Genet. 2019 Oct 29;10:982. doi: 10.3389/fgene.2019.00982 (PMC6828962; doi:10.3389/fgene.2019.00982)

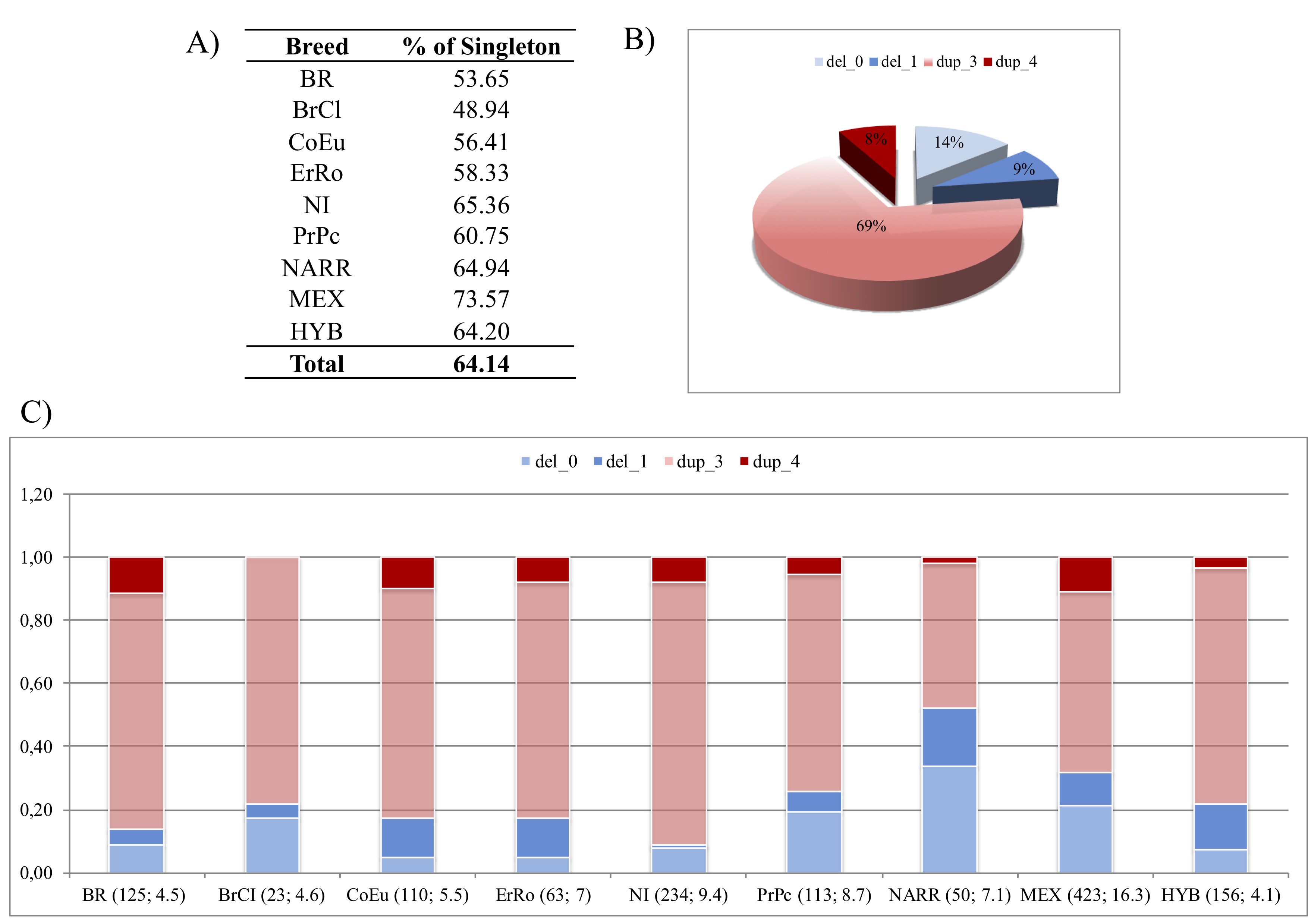

Supplement: Supplementary Figure S1 — Statistics of Singleton regions (A) Singleton % according to populations; (B) Singleton % according to state; (C) Graphical representation of Singleton % according to state and populations. [file Image_1.jpeg]

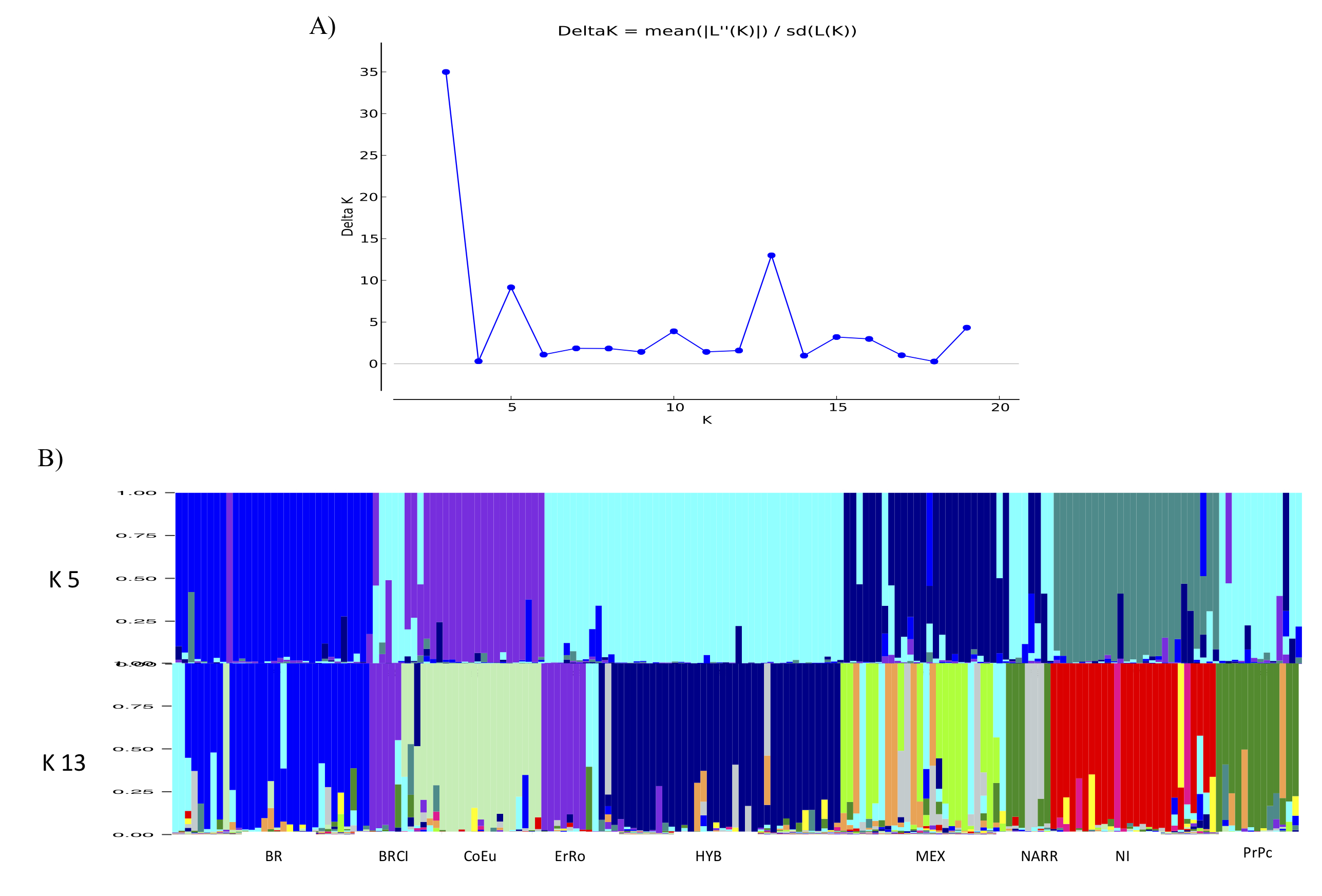

Supplement: Supplementary Figure S2 — (A) Plot representing the number of K groups that best fit the data; (B) Structure plot representing the degree of admixture among all the individuals of the nine populations considered for the two best K (K = 13 and K = 5). [file Image_2.jpeg]
